# Evaluation of the Antidepressant Activity, Hepatotoxicity and Blood Brain Barrier Permeability of Methyl Genipin

**DOI:** 10.3390/molecules21070923

**Published:** 2016-07-16

**Authors:** Xin Che, Meiyu Wang, Tian Wang, Huaying Fan, Mingyan Yang, Wenyan Wang, Hui Xu

**Affiliations:** 1 School of Pharmacy, Yantai University, Yantai 264005, China; 13792527062@163.com (X.C.); wangmei123yu@163.com (M.W.); katiefhydong@sina.com (H.F.); mingyan-123456@163.com (M.Y.); wangwenyan@luye.cn (W.W.); xuhui33@sina.com (H.X.); 2 Key Laboratory of Molecular Pharmacology and Drug Evaluation (Yantai University), Yantai 264005, China; 3 Ministry of Education, Collaborative Innovation Center of Advanced Drug Delivery System and Biotech Drugs in Universities of Shandong, Yantai 264005, China

**Keywords:** geniposide derivative, antidepressant effect, hepatotoxicity, blood–brain barrier

## Abstract

Geniposide (GE) is the main bioactive component of Gardeniae Fructus. The hepatotoxicity of geniposide limited clinical application. In order to get a new geniposide derivative that has less hepatotoxicity and still possesses the antidepressant activity, a new C-1 hydroxyl methylation derivative named methyl genipin (MG) was synthesized from geniposide. In the present study, we demonstrated that MG did not increase the liver index, alanine aminotransferase (ALT) and aspirate aminotransferase (AST). Histopathological examination suggested that no toxic damages were observed in rats treated orally with MG (0.72 mmol/kg). More importantly, a 7-day treatment with MG at 0.13, 0.26, and 0.52 mmol/kg/day could reduce the duration of immobility. It showed that the antidepressant-like effects of MG were similar to GE in the tail suspension test and the forced swim test. Furthermore, we found MG could be detected in the brain homogenate of mice treated orally with MG 0.52 mmol/kg/day for 1 day by HPLC. The area under the curve (AUC) of MG in the brain homogenate was enhanced to 21.7 times that of GE. The brain amount and distribution speed of MG were improved significantly after oral administration. This study demonstrated that MG possessed the antidepressant effects and could cross the blood–brain barrier, but had less hepatotoxicity.

## 1. Introduction

Gardeniae Fructus (Zhi-zi in Chinese) is the dried ripe fruit of the *Gardenia jasminoides* Ellis plant which belongs to the *Rubiaceae* family. It is considered to be an important traditional Chinese medicine used for the treatment of deficient dysphoria, conjunctival congestion, hemorrhage, pathopyretic ulcer, swelling, sprain, pain, and coronary artery disease [1]. Geniposide (GE) is the main bioactive component of Gardeniae Fructus, and possesses diverse beneficial biological activities, including hepatoprotective activity [2,3], neuroprotective activity [4,5,6], antidepressant [7,8,9], anti-inflammatory [10,11,12], antitumor [13], hypoglycemic [14], anti-thrombosis [15], and anti-osteoporosis properties [16].

However, hepatotoxicity has also been demonstrated in rats following oral administration of Zhi-zi, its extracts or geniposide [17,18,19]. Hepatotoxicity was manifested in phenomena such as an increase in liver enzymes, liver weight, focal necrosis, and evident inflammatory infiltration of liver cells. The possible reason was considered that geniposide was hydrolyzed into genipin by intestinal bacteria, and then genipin reacted with amino acids in vivo to gardenia blue pigment, which maybe the direct material basis for the toxicity.

**Figure 1:**
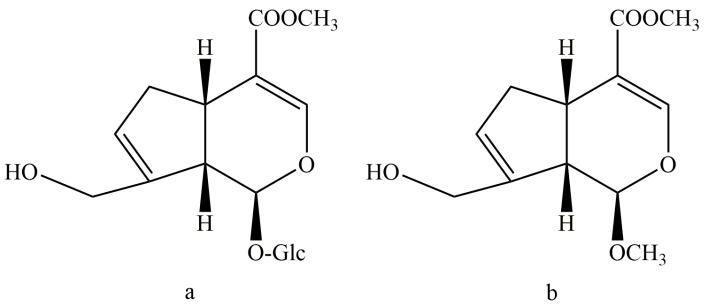
The Structure of (**a**) Geniposide (GE); and (**b**) Methyl genipin (MG).

To find a new geniposide derivative which can reduce the liver toxicity—and meanwhile keep the antidepressant activity of geniposide—we considered the structure feature by synthesizing a new C-1 hydroxyl methylation derivate from geniposide (Sichuan Taikang Pharmaceutical Co., Ltd., Sichuan, China), named methyl genipin (MG, Figure 1). The preparation method and structure identification of MG have been detailed in our previous study [20]. The key step was the acid hydrolysis and methylation reaction of GE at 65 °C for 4 h in 1 mol/L sulfuric acid-anhydrous methanol solution. The product was purified by silica gel column chromatography and semi-preparative HPLC. MG was obtained with a purity over 98%. In the present study, the hepatotoxicity, antidepressant effects, and blood–brain barrier permeability of methyl genipin were investigated for the first time.

## 2. Results

### 2.1. Effects of GE or MG on Body Weight and Liver Index in Rats

After per oral administration for 3 days at the dose of 0.72 mmol/kg (173 mg/kg), the animals in the MG group did not show significant differences in body weight and liver index compared with that of the control group (Table 1). However, the mice in the GE group showed a decrease of body weight (*p* < 0.05) and an increase of liver index (*p* < 0.01) at 0.72 mmol/kg (280 mg/kg). Furthermore, there were blue spots observed on the tails of animals, and one animal in the GE group died on the 3th day. Liver blackening and large-area necrosis were obvious after autopsies of the animals of the GE group.

### 2.2. Effects of GE or MG on Biochemical Parameters in Blood of Rats

Compared with the control group, serum alanine aminotransferase (ALT), aspirate aminotransferase (AST) and total bilirubin (TBIL) activities were increased significantly after oral administration of GE in rats at 0.72 mmol/kg (280 mg/kg). On the contrary, the levels of AST and ALT treated with MG at 0.72 mmol/kg (173 mg/kg) not only did not increase, but also reduced. Additionally, the change in TBIL levels was not obvious (Table 2).

### 2.3. Histopathological Examination

**Figure 2:**
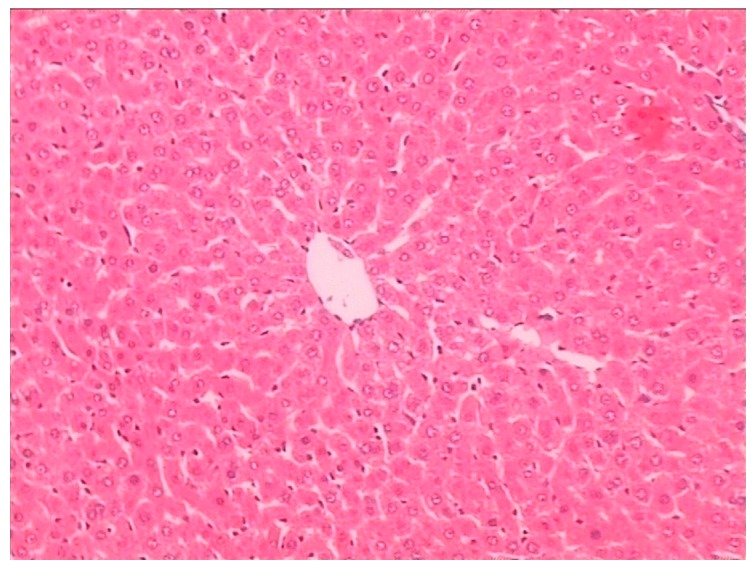
Hematoxylin and eosin (HE)-stained histological sections of rat liver in the control group (Magnification: ×400).

**Figure 3:**
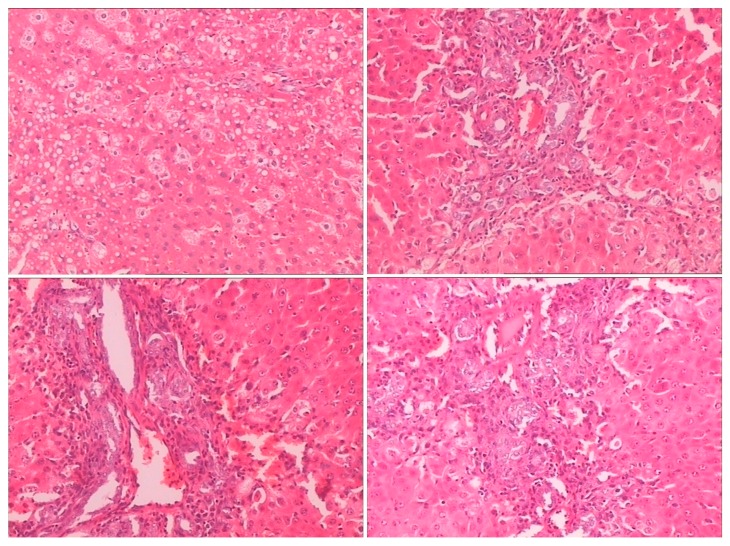
HE-stained histological sections of rat liver in the GE group (Magnification: ×400).

**Figure 4:**
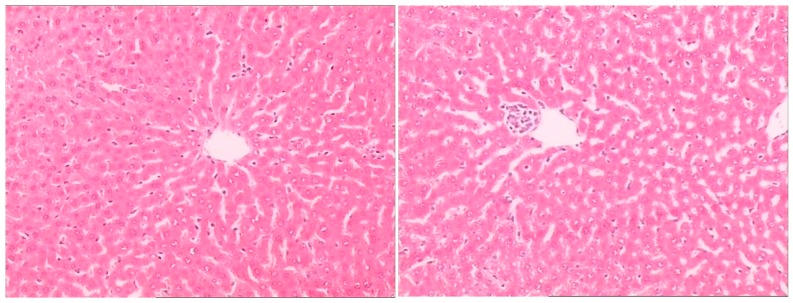
HE-stained histological sections of rat liver in the MG group (Magnification: ×400).

Histopathological examination showed there was severe toxic damage occurring in the GE group, and no injuries were observed in the MG group at the same dose of 0.72 mmol/kg. In the MG group, the livers were soft, ruddy, and shiny, with normal shape and size. There was no swelling degeneration of liver cells, or expansion of the central vein and bile duct hyperplasia in the MG group. In contrast, cell swelling degeneration, expansion of the central vein, bile duct hyperplasia, and inflammatory infiltrate were observed in the GE group. A widening of the intercellular space became wider and focal cell necrosis were also observed to occur in the GE group (Figure 2, Figure 3 and Figure 4).

### 2.4. Effects of GE or MG on Immobility Time in the Tail Suspension Test

**Figure 5:**
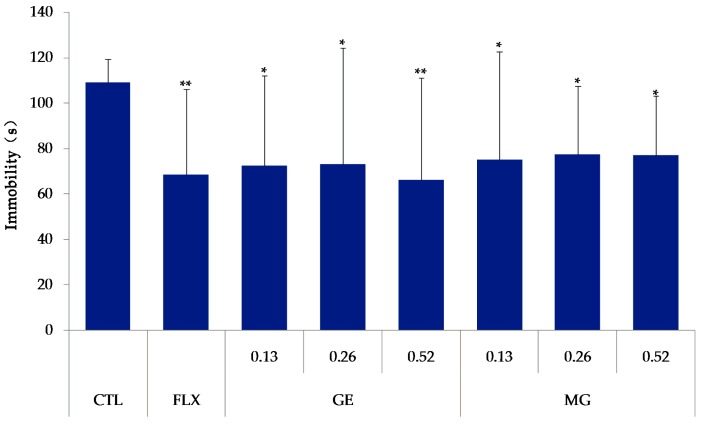
Effects of GE or MG on immobility time in the tail suspension test. The mice were treated orally with MG and GE at 0.13, 0.26, or 0.52 mmol/kg/day, and fluoxetine (FLX) at 20 mg/kg/day for 7 days. The bars indicate the mean ± SD (*n* = 10). * *p* < 0.05, ** *p* < 0.01 compared with the control group (CTL).

As shown in Figure 5, the mice were treated with fluoxetine (20 mg/kg, Changzhou Siyao Pharmaceuticals Co. Ltd., Changzhou, China), MG (0.13, 0.26, 0.52 mmol/kg) or GE (0.13, 0.26, 0.52 mmol/kg) for 7 days. Compared with the control group, all the MG, GE, and fluoxetine groups significantly decreased the immobility time (*p* < 0.01).

### 2.5. Effects of GE or MG on Immobility Time in the Forced Swim Test

**Figure 6:**
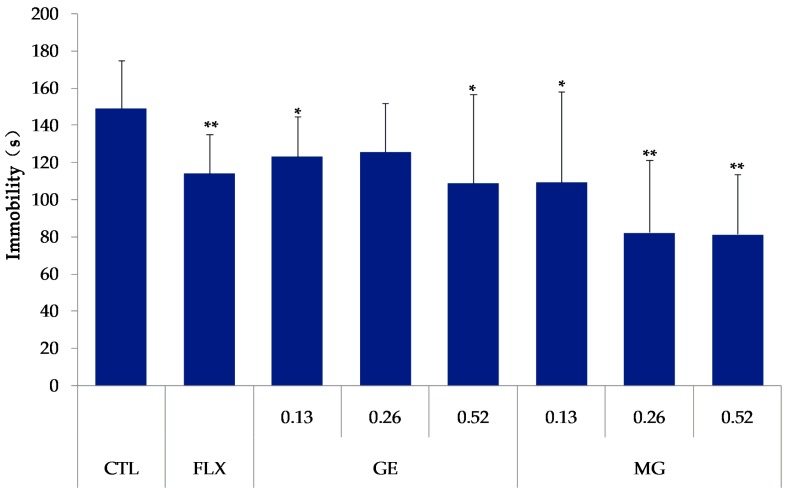
Effects of GE or MG on the immobility time in the forced swim test. The mice were treated orally with MG and GE at 0.13, 0.26, or 0.52 mmol/kg/day, and fluoxetine (FLX) at 20 mg/kg/day for 7 days. The bars indicate the mean ± SD (*n* = 10). * *p* < 0.05, ** *p* < 0.01 compared with the control group (CTL).

Compared with the control group, the immobility time of mice in the MG group and the fluoxetine group decreased (*p* < 0.05). Treatment with GE at the doses of 0.13 mmol/kg or 0.52 mmol/kg also reduced the immobility time (*p* < 0.05), Figure 6.

### 2.6. Blood–Brain Barrier Permeability

#### 2.6.1. Method of Brain Samples Qualification

**Figure 7:**
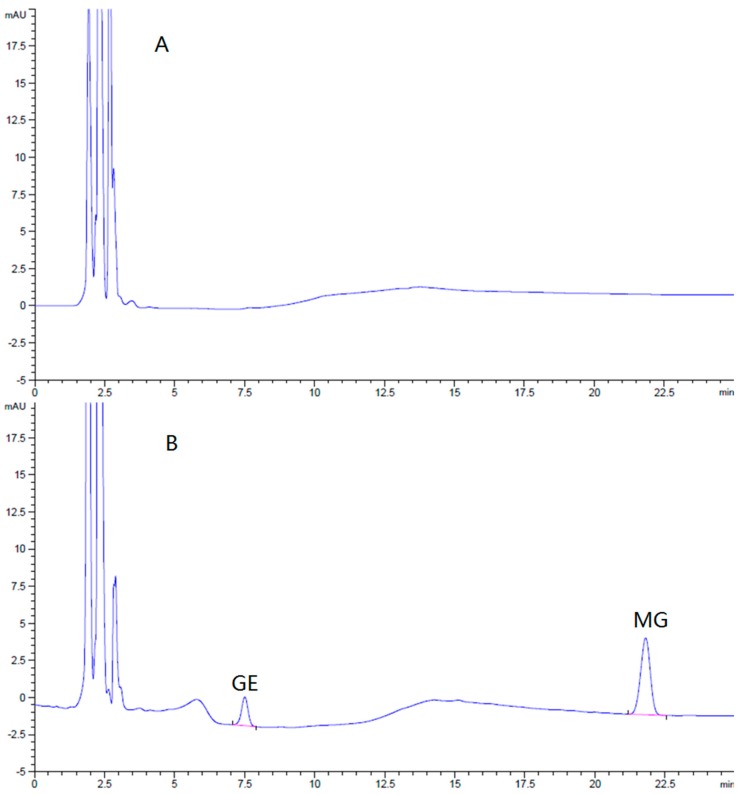

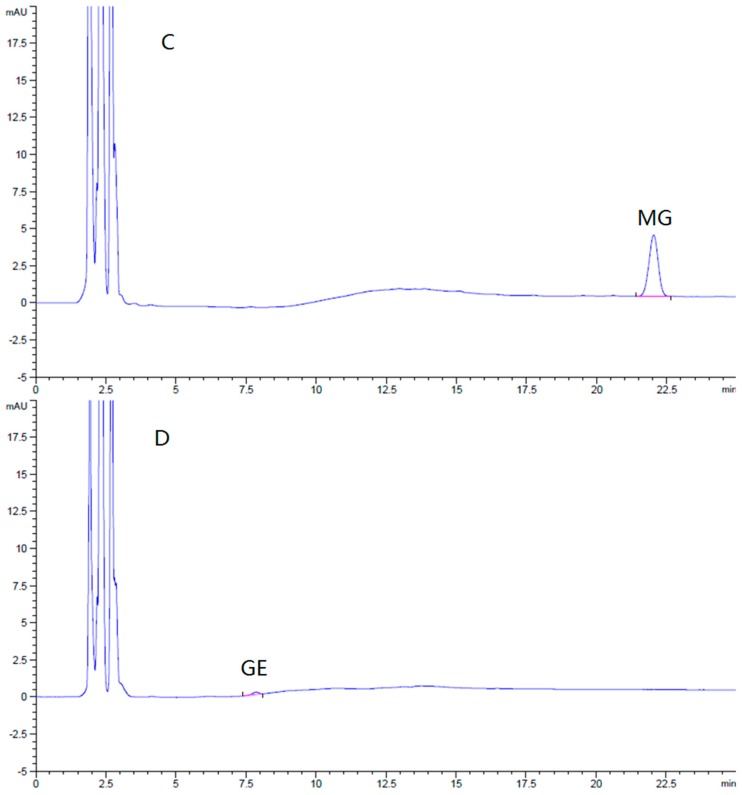
(**A**) Typical HPLC chromatograms of blank brain homogenate of mice, (**B**) blank brain homogenate of mice spiked with GE and MG; and brain homogenate sample of mice after oral administration with (**C**) MG or (**D**) GE.

The HPLC method for the assay of GE and MG in the brain was specific and efficient. There was no interference from endogenous components observed in the retention times of all the analyses in the chromatograms (Figure 7).

Brain standards covering the expected sample concentration range were prepared by spiking various quantities of GE and MG into blank brain homogenate. These calibration standards were used to validate the linearity, recovery, and precision of the analytical method. The limits of detection of GE and MG were 43 ng/g and 32 ng/g in brain (*S/N* ≥ 3), respectively. The calibration curves of GE and MG in the brain were linear**,** all in the range of 50–1500 ng/g. There was a good linearity between concentration and peak area (r > 0.999). The mean relative recoveries of GE and MG in the brain at low, middle, and high concentrations were 95.2% ± 0.30%, 95.7% ± 0.63%, 99.0% ± 1.71%, and 96.9% ± 0.17%, 96.4% ± 0.23%, and 97.7% ± 1.80%, respectively. The relative standard deviation (RSD) of intra-day precision of GE and MG were 1.6% and 0.6%, respectively, while inter-day precision were 2.4% and 4.0%. The brain sample assay method was qualified and fit for our purposes.

#### 2.6.2. Brain Pharmacokinetics

**Figure 8:**
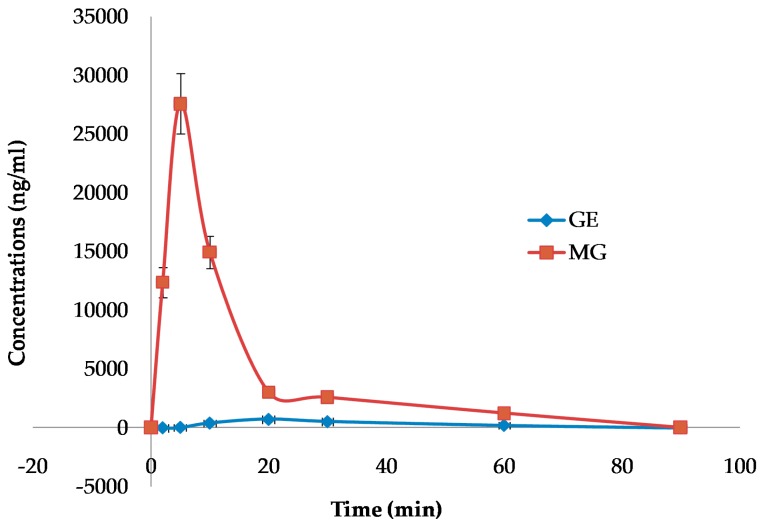
Mean brain drug concentration–time curve of mice after oral administration with GE or MG.

To further evaluate the differences in the blood–brain barrier permeability, the brain distributions of GE and MG after oral administration were detected at fixed time intervals. The concentrations of GE and MG in the brain versus time curves are displayed in Figure 8. Non-compartmental analysis of pharmacokinetic data was performed by Kinetica 4.4 software. The pharmacokinetic parameters are shown in Table 3.

The curves showed that methyl genipin, as the structure modification product, exhibited much higher distribution in the brain. The pharmacokinetic parameters indicated that C_max_ of MG in the brain after oral administration was significantly improved; the area under the curve (AUC_0–90_) and the mean residence time (MRT_last_) of MG were obviously higher than that of GE. The AUC_0–90_ for MG was enhanced to 21.7 times that of GE, and the MRT_last_ was increased to 3.29 times that of GE. The result confirmed that MG could be promptly and thoroughly transported into the brain by oral administration. The brain amount and distribution speed of MG by oral administration was improved significantly, while GE was poor and low.

## 3. Discussion

The present study aimed to explore whether the structural modification of hemiacetal hydroxyl can decrease the toxicity of geniposide, while keeping the antidepressant activity. 

It has been reported that geniposide is metabolized to genipin (aglycone) through hydrolysis by β-glucosidase (intestinal bacteria), which is in turn cleaved to a dialdehyde derivative, which reacts with polymers such as proteins, resulting in the induction of hepatotoxicty [21]. It is also known that genipin can be transformed to blue pigments by reacting with amino acids [22]. Our results indicated that there were blue spots observed on the tails, and one animal in the GE group died on that day. After autopsy, it was obvious that the liver turned black, and the kidney, stomach, and serum were blue in the animals of the GE group. However, this phenomenon was not observed in the MG group. The reason may be that MG is more stable in vivo than GE, and did not easily react with amino acids to form gardenia blue pigment.

The forced swim test (FST) and tail suspension test (TST) were well-established animal models of depression widely used to screen new potential antidepressant drugs in rats and mice [23]. The forced swimming and the tail suspension-induced state of immobility in the animals claimed to represent a condition similar to human depression, and amenable to reversal by antidepressant drugs [24]. These animal models were based on the despair or helpless behavior to some inescapable and confined space in animals, and are sensitive to various antidepressant drugs. These animal models play a role in the evaluation of antidepressant drugs. The effects of MG on the duration of immobility in the mouse FST and TST have showed that treating animals with MG orally for 7 days reduced the duration of immobility both in FST and TST significantly at the doses of 0.13, 0.26, and 0.52 mmol/kg (*p* < 0.05). However, the decrease was not dose-dependent, both in FST and TST. As a result, MG produced an antidepressant-like effect similar to the fluoxetine and GE.

MG can reduce the toxicity and play the same antidepressant activity as GE, which may provide valuable information for the application of MG in the potential treatment of depression. However, their action mode and associated mechanism(s) with anti-depression are still unclear and need to be further investigated.

Increasing the concentration of drugs in the brain is an important problem in the treatment of nervous system diseases. We hope to promote the drug crossing the blood–brain barrier by structural modification. The data in our present study demonstrated that MG from the mouse orally treated with MG can be detected by HPLC, indicating that MG can cross the blood–brain barrier of the rats. The brain amount and distribution speed of MG were improved significantly after oral administration, which may be more beneficial to the treatment of nervous system diseases.

## 4. Experimental Section

### 4.1. Animals

Male and female SD rats (200–250 g) were used in the hepatotoxicity evaluation, and male Swiss mice (18–22 g) in the antidepressant evaluation, respectively, purchased from Luye Pharma Group Co., Ltd. (Yantai, China). Mice and rats were habituated to animal facilities for 1 week before behavioral testing, and kept on a 12 h dark–light cycle, with free access to water and food. For all behavioral testing, the mice and rats were weight matched. Animal treatment and maintenance were carried out in accordance with Principles of Laboratory Animal Care (NIH Publication No. 86-23, revised in 1986) and according to the rules and ethics set forth by the Ethical Committee of Yantai University. Approval for this study was granted with the registration number: 16/ECYU-13 (dated: 16 March 2013).

### 4.2. Hepatotoxicity Evaluation of Methyl Genipin

Thirty rats were randomly divided into three groups, intragastrically administered with vehicle solution (12% alcohol solution), GE at 0.72 mmol/kg (280 mg/kg), and MG at 0.72 mmol/kg (161 mg/kg) once a day for 3 days. Drugs were dissolved in 12% alcohol solution. Blood was collected by abdominal aorta at 24 h after the last dose administration. For assessment of the hepatic damage and function analysis, serum ALT, AST, and TBIL levels were determined. The assays were performed in an AMS-18 automatic analyzer (Beijing Option Science & Technology Development Co., Ltd., Beijing, China). The livers were removed, weighed, and fixed immediately in 10% formalin, and embedded in paraffin. Tissue sections of 5 mm were made from representative regions of the organs by the conventional tissue preparation methods, and viewed under a light microscope (Olympus, Tokyo, Japan) after hematoxylin and eosin (H&E) staining. The liver sample was collected for histopathological analysis.

### 4.3. Tail Suspension Test (TST)

The procedure for TST was reported as previously described [25]. Mice were assessed in the TST, which was performed with a computerized device, allowing four animals to be tested at one time. In a chamber that was both acoustically and visually isolated, an individual mouse was suspended 50 cm above the floor by adhesive tape placed approximately 1 cm from the tip of the tail. The sessions of the animals were videotaped. Data was calculated for the total duration of immobility in the final 4 min of the 6 min test. Immobility was scored as a failure to make any struggling movements, and attempts to catch the adhesive tape or body torsions or jerks.

### 4.4. Forced Swim Test (FST)

The FST was carried out in mice, individually forced to swim in an open cylindrical container (diameter 10 cm, height 25 cm), containing 15 cm of water at 25 ± 1 °C. The sessions of the animals were videotaped. Data was calculated for the total duration of immobility in the final 4 min of the 6 min test. Each mouse was considered to be immobile when it failed to struggle and remained floating motionless in the water, making only those movements necessary to keep the nose above water. A decrease in the duration of immobility during the FST was taken as a measure of antidepressant activity [26].

### 4.5. Brain Pharmacokinetics

#### 4.5.1. Instrumentation

Instruments used in this study were a centrifuge (Anke TGL-16G, Anting Scientific Instrument Company, Shanghai, China); a vortex mixer (VDRTEX-5, Shanghai Medical University Instrument Company, Shanghai, China); and an analytical balance (METTLER-AE240). A LC-2 T HPLC system with UV detector (Shimadzu Company, Kyoto, Japan) was also used in the study.

#### 4.5.2. Chromatographic Conditions

The separation was carried out using a Diamonsil C18 column (250 × 4.6 mm, i.d., 5.0 μm) maintained at 30 °C. The mobile phase consisted of water (A) and acetonitrile (B) with a flow rate of 1.0 mL/min. GE and MG were detected at 238 nm and eluted according to the step gradient by changing the percentage of solvent B at different times, T (min)/solvent B (%) = 0/15, 5/15, 10/25, 25/25, 28/15, 35/15. Sample injection volume was 20 μL.

#### 4.5.3. In Vivo Experiments

Twenty mice were randomly divided into two groups, orally administered with GE 0.52 mmol/kg (200 mg/kg), and MG 0.52 mmol/kg (124 mg/kg) for one time. At appropriate time intervals (2, 5, 10, 20, 30, 45, 60, and 90 min), the animals were sacrificed and the brain was removed, washed by normal saline to remove excess surface blood, and were dried by filter paper. The bloodless brains were homogenized after mixing with normal saline solution two times to the weight. After centrifugation at 12,000 rpm for 10 min, 800 μL acetonitrile (the brain homogenate was 200 μL) was added in the supernatant to precipitate proteins in the sample. After vortexing for 3 min, the solution was centrifuged at 12,000 rpm for 5 min. Then, the supernatant was removed and evaporated under air stream in a water bath at 50 °C. The residue was dissolved in 100 μL of methanol, vortexed for 30 s, and centrifuged at 12,000 rpm for 5 min. A 20 μL aliquot of supernatant was injected into the HPLC system for analysis.

### 4.6. Statistical Analysis

Data analyses were performed using one-way analysis of variance (ANOVA), followed by Tukey’s *post-hoc* test, using Prism 5 (GraphPad Software, Inc., San Diego, CA, USA) for multigroup comparisons. All data are presented as the mean ± standard error (SEM). Significance was set at *p* < 0.05.

## 5. Conclusions

In the present study, we demonstrated that even if MG was orally administered at large dose, there was no hepatotoxicity observed. Meanwhile, the antidepressant-like effects of MG was similar to GE in the tail suspension test and the forced swim test of mice at 0.13, 0.26, and 0.52 mmol/kg/day orally administered for seven days. Furthermore, the AUC of MG in the brain homogenate was enhanced to 21.7 times that of GE, detected in the brain homogenate by HPLC of mice at 0.52 mmol/kg/day orally administered for 1 day. The brain amount and distribution speed of MG by oral administration were improved, while GE was poor and low. For the first time, this paper evaluated the hepatotoxcity, antidepressant effects, and blood–brain barrier permeability of MG. However, more studies are needed to further investigate the pharmacological activity and associated mechanism of MG as a novel antidepressant. 

## Figures and Tables

**Table 1:** Effects of GE or MG on body weight and liver index after oral administration in rats.

Group	Dosage (mmol/kg)	N	Body Weight before Administration/g	Body Weight after Administration/g	Liver Index
Control	—	10	265.1 ± 18.9	245.1 ± 16.3	3.1 ± 0.5
Geniposide	0.72	10 (1)	251.4 ± 9.7	226.3 ± 8.1 *	4.4 ± 0.5 **
Methyl genipin	0.72	10	259.4 ± 13.8	243.0 ± 11.8	2.7 ± 0.2

* *p* < 0.05; ** *p* < 0.01; () Number of deaths.

**Table 2:** Effects of GE or MG on biochemical parameters in blood of rats. ALT: alanine aminotransferase; AST: aspirate aminotransferase; TBIL: total bilirubin.

Group	Dosage (mmol/kg)	N	ALT/U·L^−1^	AST/U·L^−1^	TBIL/μmol·L^−1^
Control	—	10	65.8 ± 10.8	179.4 ± 37.3	6.7 ± 4.2
Geniposide	0.72	10 (1)	1304.6 ± 14.7 **	646.4 ± 15.4 **	33.1 ± 9.8 **
Methyl genipin	0.72	10	50.0 ± 8.6 *	118.3 ± 19.7 **	10.2 ± 4.0

* *p* < 0.05, ** *p* < 0.01; () Number of deaths.

**Table 3:** Main brain pharmacokinetic parameters of GE and MG in mice after oral administration. AUC_0–90_: Area under the curve; MRT_last_: mean residence time.

Group	Parameters			
	T_max_ (min)	C_max_ (ng/mL)	AUC_0–90_ (ng/mL·min)	MRT_last_ (min)
MG	5 **	27592.7 ± 2536.7 **	332031.6 ± 28741.2 **	15.3 ± 0.6 **
GE	20	735.2 ± 64.9	23636.7 ± 2248.8	29.8 ± 2.6

Data are expressed as mean ± SD (*n* = 5). ** *p* < 0.01 vs. the GE group.
